# Ezrin overexpression predicts the poor prognosis of gastric adenocarcinoma

**DOI:** 10.1186/1746-1596-7-135

**Published:** 2012-10-05

**Authors:** Jingchun Jin, Tiefeng Jin, Meiling Quan, Yingshi Piao, Zhenhua Lin

**Affiliations:** 1 Department of Pathology, Yanbian University Medical College, Yanji-City, 133002, Jilin-Prov., P.R. China; 2 Cancer Research Center, Yanbian University, Yanji-City, 133002, Jilin-Prov., P.R. China; 3 Department of Pathophysiology, Yanbian University Medical College, Yanji-City, 133002, Jilin-Prov., P.R. China; 4 Department of Internal Medicine, Yanbian University Affiliated Hospital, Yanji-City, 133000, Jilin-Prov., P.R. China

**Keywords:** Gastric adenocarcinoma, Ezrin, Tissue microarray

## Abstract

**Background:**

Ezrin is a cytoskeletal protein that is involved in tumor growth and invasion. It has been suggested that Ezrin expression plays an important role in tumor metastasis. This study is aimed to investigate the clinicopathological significance of Ezrin overexpression in gastric adenocarcinomas.

**Methods:**

Ezrin protein expression was examined by immunohistochemistry in 26 normal gastric mucosa, 32 dysplasia, and 277 gastric adenocarcinomas. The relationship between Ezrin expression and the clinicopathological features of gastric cancers was analyzed. In addition, a gastric cancer cell line, MKN-1, was also used for immunofluorescence staining to evaluate the distribution of Ezrin protein.

**Results:**

Ezrin protein located in the cytoplasm and/or membrane in the migrating gastric cancer cells, and it mainly concentrated at the protrusion site; however, only cytoplasmic distribution was observed in the non-migrating cancer cells by immunofluorescence staining. The positive rate of Ezrin protein expression was significantly higher in gastric adenocarcinoma and dysplasia compared with that in the normal gastric mucosa. Moreover, expression frequency of Ezrin protein increased significantly in lymph node metastasis and late clinical stages. Consistently, strong expression of Ezrin was significantly correlated with poor prognosis of gastric cancer.

**Conclusion:**

The detection of Ezrin expression can be used as the marker for early diagnosis and prognosis of gastric adenocarcinoma.

**Virtual Slides:**

The virtual slide(s) for this article can be found here: http://www.diagnosticpathology.diagnomx.eu/vs/2303598677653946

## Introduction

Gastric cancer is one of the most fatal malignant tumors worldwide. The poor prognosis is associated with extensive local invasion and/or regional lymph node metastasis [1]. Local recurrence remains the cause of cancer-related deaths after resection in a substantial proportion of patients with gastric cancer. Therefore, establishing reliable criteria to predict recurrence and to identify the tumors is of great interest not only for understanding the molecular and cellular processes involved in tomorigenesis, but also for searching the possible new therapeutic molecular targets [2].

Tumor metastasis starts with breakdown of epithelial integrity, followed by malignant cells invading into the surrounding stroma and lymphovascular space, by which tumor cells travel to distant target organs [3,4]. Cell adhesion molecules and actin cytoskeleton play a crucial role in tumor metastasis [5,6]. The primary mechanism for most types of cell migration is the actin cytoskeleton remodeling [7]. The cytoskeletal protein Ezrin is a member of the Ezrin-Radixin-Moesin (ERM) family which is linked to aggressive tumor behavior by involving all stages of tumor metastasis [7,8] including cell adhesion, survival, motility, and signal transduction [9-11].

Recent publications showed that Ezrin is strongly expressed in a variety of invasive cancers, including osteosarcoma, melanoma, soft tissue sarcoma, pancreatic carcinoma, hepatocellular carcinoma and gastric and breast cancers [2,11-17]. There is accumulating evidence suggesting that Ezrin is a metastatic determinant and a key component in tumor metastasis, however, its exact role in gastric cancer is still unknown. Bal et al. [18] reported that there was a negative correlation between Ezrin and lymph node metastasis, lymphovascular space invasion, and perineural invasion in all gastric carcinomas, but was not statistically significant (P > 0.05), while no association with depth of invasion, tumor location, tumor size and distant metastasis (P > 0.05). However, Zhao et al. [19] and Li et al. [20] reported that positive expression of Ezrin correlated with age, tumor size, location, differentiation stage, depth of invasion, vessel invasion, lymph node and distant metastasis, and TNM stage (P < 0.05). In present study, we therefore aimed to investigate the Ezrin protein expression in human gastric adenocarcinoma and its precancerous lesions, and to explore the exactly relation of Ezrin expression to the clinical outcomes and the histological parameters of gastric cancers.

## Materials and methods

### Clinical samples

Total 335 tissue samples, including 277 cases of gastric adenocarcinomas, 32 cases of dysplasia and 26 of normal gastric tissues, were collected from Shanghai Outdo Biotech Co. Ltd. (Outdo Biotech) and Department of Pathology, The Third Affiliated People’s Hospital of Shanghai Jiaotong University. All tissues were routinely fixed in 10% buffered formalin and embedded in paraffin blocks. The study protocol was approved by the institutional review board of Yanbian University Medical College.

The pathological parameters, including age, gender, histological types, differentiation, the presence of nodal metastasis, clinical stage and disease free survival, were carefully reviewed in all of 277 gastric adenocarcinomas. The patients’ age ranged from 36 to 78 yr with a mean age of 51.7 yr. The male to female ratio was 164:113. Of the 277 gastric adenocarcinomas encompassed 39 cases of TNM stage 0, 98 cases of TNM stage I (TNM stage IA = 47, TNM stage IB = 51), 75 cases of TNM stage II, 59 cases of TNM stage III, and 6 cases of TNM stage IV. In which, 85 cases were well differentiated adenocarcinoma, 103 cases as moderately differentiated, 59 cases as poorly differentiated, 5 cases as undifferentiated, 9 cases as signet ring cell carcinomas, and 16 cases as mucinous adenocarcinoma. For the Lauren types, 117 cases were intestinal type, 139 cases as diffuse type, and 21 cases as mixed types. TNM staging was assessed according to the staging system established by the American Joint Committee on Cancer (AJCC) [21]. Of the 277 gastric adenocarcinomas, 151 cases were lymph node (LN) metastasis negative, and 126 cases were LN meatastasis positive. In total 277 of gastric adenocarcinomas, 54.9% (152/277) of cases were more than three years of disease free survival. Additionally, the normal gastric mucosa tissues were obtained from the resection margins of radical specimen of gastric cancer.

### Immunofluorescence staining for Ezrin protein in cancer cells *in vitro*

Gastric cancer cell line MKN-1 was grown on coverslips to 100% confluence, and then continued to culture with FBS free medium for 24 hours after being scratched by a new 200 μl pipette tip for searching the migrating cells. The cells were then fixed with 4% paraformaldehyde for 10 minutes and permeabilized with 0.5% TritonX-100 for 10 minutes after 24 hours. Blocking was performed with 3% Albumin Bovine V (A8020, Solarbio, Beijing, China) for one hour at the room temperature. After washing with PBS, cells were incubated with antibody against Ezrin (1:100, #3145, Cell Signaling Technology, Boston, USA) for two hours, and followed the incubation by Alexa Fluor^®^488 goat anti-rabbit IgG (H + C) (A11008, Invitrogen, USA) for one hour at room temperature. After washing with PBS, the cells were counterstained with 49-6-diamidino-2-phenylindole (DAPI) (C1006, Beyotime, Shanghai, China), and the coverslips were mounted with Antifade Mounting Medium (P0126, Beyotime, Shanghai, China). Finally, the immunofluorescence signals were visualized and recorded by Leica SP5II confocal microscope.

### Immunohistochemistry for Ezrin protein in paraffin-embedded tissues

Dako LSAB kit (Dako, Glostrup, Denmark) was used for immunohistochemistry. And the serial 4 μm-thick tissue sections were prepared on silane-coated slides (Sigma, St Louis, MO, USA), and deparaffinized, rehydrated and incubated with 3% H_2_O_2_ in methanol for 5 minutes at room temperature to eliminate endogenous peroxidase activity. The antigen was retrieved at 95°C for 20 minutes by placing the slides in 10 mM sodium citrate buffer (pH 6.0). The slides were then incubated with primary antibody Ezrin (1:50, #3145, Cell Signaling Technology, Boston, USA) at 4°C for overnight. After incubation at room temperature for 30 minutes with biotinylated secondary antibody, the slides were incubated with streptavidin-peroxidase complex at room temperature for 30 minutes. Immunostaining was developed by using chromogen, 3,3'-diaminobenzidine, and counterstained with Mayer's hematoxylin. Rabbit IgG isotope used as the control and the result is negative. Also, the positive tissue sections were processed omitting the primary antibody as negative controls.

### Interpretation of immunohistochemical staining

**Figure 1:**
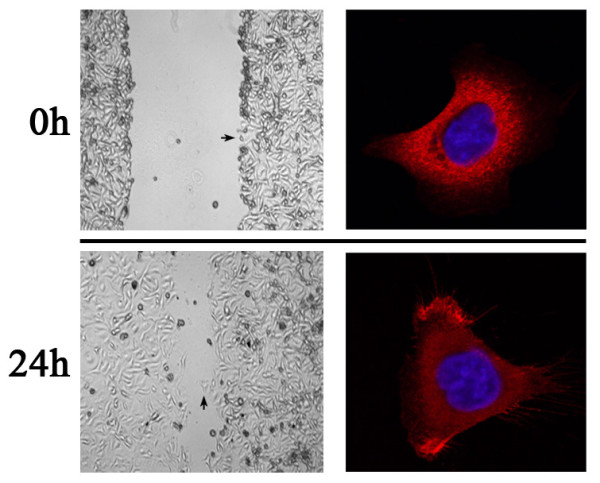
**Immunofluorescence staining for Ezrin protein in cultured MKN-1 cells (red for Ezrin protein & blue for DAPI).** Ezrin protein located in the cytoplasm and membrane in cultured MKN-1 migrating gastric cancer cells, and it mainly concentrated at the membranous protrusion site (Figure 1, 24 h); however, Ezrin protein only located at the cytoplasm of non-migrating cancer cells (Figure 1, 0 h).

All slides were scored independently by two investigators (Lin Z and Piao Y) being blinded to all clinical data. The interpretation criteria were described previously by Elzagheid A et al. [22]. Briefly, lymphocytes served as a reference for strong immunoreactivity (Figure 1), and the immunoreactivity was graded into four categories: +++ (score 3) = similar to the lymphocyte staining; ++ (score 2) = less than +++; + (score 1) = distinguishable from the background staining; and – (score 0) = completely negative. Only the cytoplasmic and membranous expression was considered as positive staining and the strong positive means ‘++’ and ‘+++’ positive cells. 

### Statistical analysis

Statistical analysis was performed using the Chi-square (*x*^2^-test) test and Mean-Whitney test of SPSS software program for windows, version 17.0 (SPSS, Chicago, USA). P**-**value less than 0.05 considered significant.

## Results

### The characteristics of Ezrin protein localization and distribution

**Figure 2:**
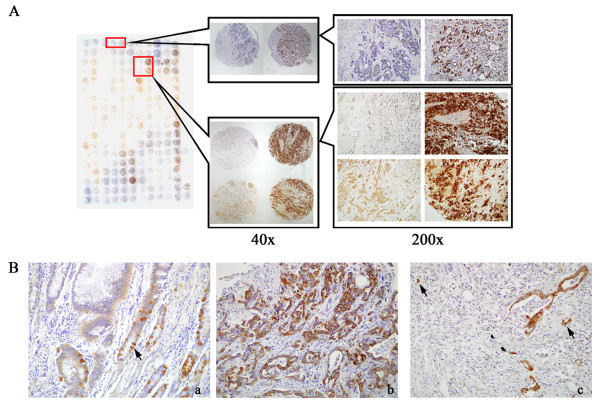
**Immunoreactivity for Ezrin protein in gastric lesions.** Immunohistochemical staining for Ezrin protein in the tissuearray of gastric adenocarcinoma (**A**). Scattered positive cells (arrows) for Ezrin protein were seen at the cytoplasm of basal reserve cells in gastric normal mucosa (**B**-**a**) (200×). The gastric cancer cells showed strongly and diffusely positive staining for Ezrin protein (**B**-**b**) (200×). Single scattered cancer cells and invasive cancer loci at the stroma showed intense immunoreactivity for Ezrin protein (arrows) (**B**-**c**) (200×).

To observe the localization of Ezrin protein in migrating and non-migrating cancer cells, the cultured MKN-1 gastric cancer cells were scratched by a new 200 μl pipette tip (Figure 1), and then the immunofluorescence staining for Ezrin protein was done. It was found that Ezrin protein located at the cytoplasm and/or membrane in the migrating MKN-1 cells, and mainly concentrated at the protrusion site; however, only cytoplasmic distribution was observed in the non-migrating MKN-1 cells by immunofluorescence staining (Figure 1). For the tissue sections, diffusely and strongly positive signals for Ezrin protein was detected in the cytoplasm of gastric cancer cells, however negative or scattered positive cells, mainly basal reserve cells, was observed in the normal gastric epithelia by immunohistochemistry. Interestingly, single scattered cancer cells or invasive cancer loci at the stroma frequently showed strongly and most intense immunoreactivity for Ezrin protein (Figure 2).

### Correlation between Ezrin protein overexpression and clinical parameters of gastric cancers

**Figure 3:**
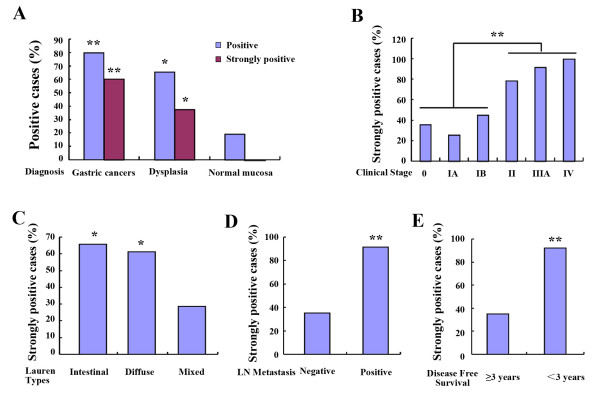
**Ezrin protein showed significantly higher positivity in gastric adenocarcinoma and dysplasia compared with the adjacent normal gastric mucosa (A), and its expression level was significantly higher in the late stage (Stage II, Stage III, and Stage IV) of gastric cancers than it in the early stage (Stage 0, Stage IA, and Stage IB) cases (B).** Moreover, strongly expression of Ezrin protein was detected in Lauren intestinal and diffuse type of gastric adenocarcinomas than it in the mixed type cases (**C**). Ezrin protein overexpression showed closely correlation to the metastatic status (**D**) and disease free survival of gastric adenocarcinoma (**E**).

Ezrin protein showed higher positivity in gastric adenocarcinoma (score 1, 79.8%, 221/277; score 2, 60.6%, 168/277) compared with the adjacent normal gastric mucosa (score 1, 19.2%, 5/26; score 2, 0, 0/26). Also, Ezrin protein was strongly positive in gastric dysplasia (score 1, 65.6%, 21/32; score 2, 37.5%, 12/32) on immunohistochemistry, which was also significantly higher than normal gastric tissues (score 1, 19.2%, 5/26; score 2, 0%, 0/26). Similarly, Lauren intestinal (65.8%, 77/117) and diffuse (61.2%, 85/139) types of gastric cancer also determined strongly expression rate of Ezrin protein compared to the mixed type (28.6%, 6/21) cases (P < 0.05). (Figures 2 &3, Tables 1 &2).

**Table 1:** Ezrin protein expression in gastric adenocarcinoma

**Diagnosis**	**Number of cases**	**Positive cases**	**Positive rate (%)**	**Strongly positive rate (%)**
		**- + ++ +++**		
Gastric adenocarcinoma	277	56 53 102 66	79.8%**	60.6%**
Dysplasia	32	11 9 12 0	65.6%*	37.5%*
Normal mucosa	26	21 5 0 0	19.2%	0

Compared with normal mucosa *P < 0.05, **P < 0.01. Strongly positive: ++ and +++.

**Table 2:** Relationship between Ezrin protein overexpression and clinicopathological features of gastric adenocarcinoma

**Clinical features**	**No. of cases**	**Strongly positive cases (%)**	**P-value**
**Age**			
<50	96	60 (62.5%)	NS
51-69	181	92 (50.8%)	
≥70	29	16 (55.2%)	
**Gender**			NS
Male	164	102 (62.2%)	
Female	113	66 (58.4%)	
**Lauren Types**			<0.05, ***a***
Intestinal type	117	77 (65.8%)	
Diffuse type	139	85 (61.2%)	
Mixed type	21	6 (28.6%)	
**WHO’s Histological Types**			NS
Well-diff. ade.	85	42 (49.4%)	
Moderately-diff. ade.	103	54 (52.4%)	
Poorly-diff. ade.	59	50 (84.7%)	
Undifferentiated ade.	5	3 (60.0%)	
Signet ring cell carcinoma	9	5 (55.6%)	
Mucinous ade.	16	14 (87.5%)	
**LN Metastasis**			<0.01
Negative	151	53 (35.1%)	
Positive	126	115 (91.3%)	
**Clinical Stage**			<0.05, ***b***
0	39	14 (35.9%)	
IA	47	12 (25.5%)	
IB	51	23 (45.1%)	
II	75	59 (78.7%)	
IIIA	59	54 (91.5%)	
IV	6	6 (100%)	
**Disease Free Survival**			<0.01
≥3 years	152	53 (34.9%)	
<3 years	125	115 (92.0%)	

ade.: Adenocarcinoma; diff.: differentiated; NS: not significant. * Strongly positive: ++ and +++.

***a***: Intestinal & Diffuse types *vs* Mixed type.

***b***: Stage 0 + Stage IA + Stage IB *vs* Stage II + Stage IIIA + Stage IV.

Additionally, Ezrin protein overexpression was significantly correlated with the lymph node metastasis of gastric adenocarcinoma. The strongly positive rates of Ezrin were 35.1% (53/151) and 91.3% (115/126) in non-metastatic and metastasic carcinoma of stomach, respectively (P<0.01). For the TNM clinical stages, Ezrin positive rate was only 35.8% (49/137) in early clinical stage (35.9% in Stage 0, 25.5% in stage IA and 45.1% in stage IB) of gastric cancer, however significantly higher in late stage cases (85.0%, 119/140) (78.7% in Stage II, 91.5% in stage III, and 100% in stage IV), and the difference was statistically significant (P<0.05). Also, the strongly positive rate of Ezrin protein expression was significantly higher in <3 years disease free survival cases (92.0%, 115/125) than it in ≥3 years disease free survival cases (34.9%, 53/152) (P < 0.01). However, Ezrin protein expression level was not correlated with the patient age, gender, histological type status of gastric adenocarcinoma (P>0.05) (Figure 3, Table 2).

## Discussion

Gastric cancer is the one of most common malignant tumor worldwide. Despite effective control of the primary tumor and both neoadjuvant and adjuvant chemotherapy, the development of metastases is still the common cause of death in gastric cancer patients [23,24]. The development of new and effective treatments based on the well understanding of metastasis biology is needed.

Human Ezrin gene maps to chromosome 6q25.2-q26 and the total length of mRNA is 3166 bp, encoding 585 amino acids. Ezrin has been shown to bind directly to PI3K and influence many signaling pathways that affect cellular functions related to tumorigenesis and metastasis, including MAPK-ERK1/2, PI3K-Akt and Rho pathways. Recently, increasing reports also showed that the critical functions of Ezrin are the regulation of cell shape, motility, adhesion and signal transduction, all of which are important for tumor development and progression [25].

Wang et al. [26] reported that the inhibition of Ezrin expression clearly inhibited the migration and invasion of the human gastric cancer cell line SGC-7901, and increased both cell adhesion and sensitivity to camptothecin-induced apoptosis. Overexpression of Ezrin also promoted cell protrusion, microvillus formation, anchorage-independent growth, motility and invasion in the pancreatic cancer cell line, MiaPaCa-2 [14]. Since then, Ezrin expression has been linked to clinical outcome and prognosis in many cancer types including osteosarcoma, pancreatic carcinoma, hepatocellular carcinoma, and breast carcinoma [14-17].

As a member of ERM protein family, Ezrin functions as a linker protein connecting the actin cytoskeleton (Ezrin C-terminus) to integral plasma membrane proteins (Ezrin N-terminus) [27]. It is proposed that Ezrin exists in a dormant form in which the C-terminal tail binds to and masks the N-terminal FERM domain [28]. Therefore, amino-terminal Ezrin interactions are critical in determining not only the repertoire of proteins Ezrin can interact with but also the corresponding cellular functions that may be positively or negatively affected [27]. This linkage to the cell membrane allows the cells to physically engage and potentially sense the tumor microenvironment [27,28]. Elzagheid et al. [22] reported that Ezrin was predominantly expressed at the apical cell membrane in a polarized fashion in normal colonic epithelium. In contrast, Ezrin expression in the cancer cells was typically cytoplasmic. In the present study, Ezrin protein was found to locate in the cytoplasm and/or membrane in the migrating gastric cancer cells *in vitro*, and it mainly concentrated at the protrusion site of MKN-1 gastric cancer cells; however, Ezrin protein located only in the cytoplasm in non-migrating cells *in vitro* by immunofluorescence staining (Figure 1). By the immunohistochemistry, the diffusely and strongly positive signals for Ezrin protein was detected in the cytoplasm of gastric cancer cells; however, negative or scattered positive cells (mainly basal reserve cells) was observed in the cytoplasm of normal gastric epithelia, indicating that the subcellular distribution of Ezrin was predominantly cytoplasmic distribution in non-migrating cancer or normal cells, but mainly membranous distribution in the migrating cells *in vitro*. This is consistent with the previous reports in other epithelial human tumors. However, apical localization of Ezrin protein was seen neither in gastric carcinoma nor in normal gastric epithelia by immunohistochemistry. More interestingly, scattered single cancer cells at the stroma frequently showed stronger and most intense immunoreactivity in this study, and similar observations was reported previously in colorectal cancers by Elzagheid et al. [22] and endometrioid carcinomas by Köbel et al. [29] and Yasuoka et al. [30]. These data indicated that Ezrin might be essential for the processes of gastric cancer cells, including the determination of cell shape, polarity and formation of surface structures, motility, and integration of membrane transport with signaling pathways. But the detailed mechanism needs to be explored by the further study.

Recently, it has been shown that Ezrin plays a pivotal role in the progression of gastrointestinal carcinoma [2,18-20,22]. Elzagheid et al. [22] reported that Ezrin may play a role in colorectal cancer progression and that Ezrin expression might provide clinically valuable information in predicting the biological behavior of colorectal cancer. Zhao et al. [19] reported that overexpression of Ezrin promoted gastric cancer cell invasion, whereas inactivating Ezrin function with small interference RNA caused reduced cell invasion, indicating a potential role of Ezrin in regulating the progression to invasive gastric cancer. In the present study, 277 cases of gastric adenocarcinomas, 32 of dysplasia, and 26 of normal gastric mucosa were investigated, and it was found that Ezrin expression was significantly up-regulated in gastric cancers and dysplasia compared with normal gastric mucosa, however no difference was found between gastric cancer and dysplasia, indicating that Ezrin protein overexpression could be used as the early diagnostic marker for gastric cancer and its precancerous disease.

It is well known that TNM staging system according to The American Joint Committee on Cancer (AJCC)/International Union against Cancer (UICC) produced the most reliable system for predicting the survival of patients. Furthermore lymphatic and vascular invasion were also considered as poor prognostic indicators [31]. Limited reports suggest that Ezrin may be a useful prognostic and survival indicator for gastric cancers. Zhao et al. [19] and Fan et al. [2] demonstrated that Ezrin was required for the invasion of gastric cancer cells. However, Bal et al. [18] reported that no statistically significance was found about the correlation of ezrin overexpression and lymph node metastasis, lymphovascular space invasion, and distant metastasis. Here we found that the strongly positive rate of Ezrin protein expression was significantly higher in metastatic gastric cancer (91.3%) than it in non-metastatic cancer cases (35.1%) (P < 0.01). For the TNM clinical stages, the strongly positive rate of Ezrin was lower in Stage 0 (35.9%) and stage I (Stage IA: 25.5%; Stage IB: 45.1%) compared with Stage II (78.7%), Stage III (91.5%) and Stage IV (100%), the difference was statistically significant (P < 0.05), demonstrating that Ezrin protein overexpression was strongly correlated with the lymph node metastasis and clinical stage of gastric cancers. Additionally, Li et al. [20] reported that for 436 gastric cancer patients with stage I, II or III disease, the 5-year survival rate for those with high Ezrin expression were significantly lower than in patients with low expression. Zhao et al. [19] also reported that the survival rate of patients with Ezrin or c-Met positive gastric cancers were significantly lower than those in patients with Ezrin or c-Met negative tumors (P<0.05). However, here we also found that the strongly positive rate of Ezrin protein expression was significantly higher in <3 years disease free survival cases (92.0%) than it in ^3^3 years disease free survival cases (34.9%) (P<0.01). All above data strongly indicated that Ezrin could be regarded as a potential prognostic factor in gastric cancers.

Moreover, Lam et al. [32] reported that among 150 gastric cancer cases, 33 (22.0%) cases showed low Ezrin expression, 92 (61.3%) cases showed moderate Ezrin expression and 25 (16.7%) cases showed high Ezrin expression. Ezrin expression was associated with Lauren type and differentiation but not correlated with the patients’ age and gender. However, Li et al. [20] reported that Ezrin positive expression is correlated with age, tumor size and location, grading and poor prognosis. In the present study, higher strongly expression rate of Ezrin protein was detected in intestinal type (65.8%) and diffuse type (61.2%) of gastric cancer than that in the mixed type (28.6%) cases. But Ezrin protein expression level was not correlated with the patient age, gender, WHO’s histological type status of gastric carcinomas (P>0.05). This is consistent with Lam’s report, but it needs the further study to verify.

All above data point to the importance of Ezrin not only as a useful marker of early diagnosis and prognosis but also as a potential therapeutic target in gastric adenocarcinoma. The high frequency of Ezrin expression suggests a central role in gastric cancer biology, though the further study needs to be investigated for exploring the mechanism in detail. In summary, the detection of Ezrin protein expression could be used as an early diagnostic marker of gastric cancer and its precancerous disease, and Ezrin overexpression could predict the poor prognosis of gastric adenocarcinoma, suggesting that Ezrin might be a potential molecular target for gastric adenocarcinoma therapy.

## Competing interests

Authors declare no conflict of interests.

## Author’s contributions

JJ and JT participated in study conception, design, case selection and immunohistochemical staining. QM and PY carried out immunofluorescence staining and data collection. PY and LZ performed data analysis and writing the manuscript. All the authors read and approved the final manuscript.
